# Nanoscale Graphene‐Based Ballistic Rectifiers for Detection of High Terahertz Frequency Optical Signals

**DOI:** 10.1002/smsc.202500654

**Published:** 2026-03-29

**Authors:** Lili Shi, Leonardo Viti, Kenji Watanabe, Takashi Taniguchi, Miriam Serena Vitiello

**Affiliations:** ^1^ NEST, CNR‐NANO and Scuola Normale Superiore Pisa Italy; ^2^ National Institute for Materials Science Tsukuba Japan

**Keywords:** ballistic transport, graphene, photodetectors, terahertz

## Abstract

Graphene exhibits the longest carrier mean free path of any known electronic material, yet only a few device concepts have successfully leveraged this exceptional property. Here, we present a ballistic graphene rectifier capable of operating at frequencies up to 3 THz, significantly extending the limits of direct current (DC) generation through rectification in two‐dimensional materials. By engineering asymmetric nanojunction geometries in high‐mobility monolayer graphene encapsulated in hBN, we harness ballistic transport over >100 nm length scales to achieve efficient rectification without relying on p–n junctions or Schottky barriers. The devices exhibit robust rectified signals, with voltage responsivities of 100 V/W, 20 pW/Hz^1/2^ noise‐equivalent powers at room temperature, and minimum detectable powers of 30 nW, outperforming conventional semiconductor‐based rectifiers in the same frequency range. Our results establish a new pathway for passive THz detection and signal processing, highlighting the potential of graphene nanodevices for next‐generation high‐frequency technologies.

## Graphical abstract

Ballistic graphene rectifier capable of operating at frequencies up to 3 THz, engineered through asymmetric nanojunction geometries in high‐mobility monolayer graphene encapsulated in hexagonal boron nitride

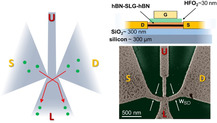

## 1 Introduction

Ballistic rectifiers (BRs) [1, 2] are nanoscale electronic devices that exploit the principles of ballistic transport to convert alternating current (AC) signals into direct current (DC) without relying on conventional *p–n* junctions or Schottky barriers. In these devices, electrons propagate through the structure with minimal scattering, following trajectories defined primarily by the device geometry rather than by the electric fields or doping profiles. This allows BRs to operate with extremely high speed and low noise, and with extremely small dimensions—making them attractive for high‐frequency signal processing, sub‐millimeter–wave detection, and future nano‐electronic applications.

By leveraging the high carrier mobility of state‐of‐the‐art two‐dimensional (2D) materials—2D electron gases in semiconductor heterostructures [3], or graphene [4]‐ it is possible to engineer BRs that achieve strongly nonlinear electrical transport through a narrow (≤100 nm) junction. Specifically, when the electron mean free path exceeds the characteristic device dimensions, transport shifts from diffusive to ballistic, with specular reflections off the device boundaries becoming the dominant transport mechanism [5, 6]. In the absence of scattering, charge carriers can move freely without interference from phonons or impurities. In this ballistic regime, the geometry of the nanoscale device itself can be engineered to impose nonlinear transport through it [7].

**FIGURE 1:**
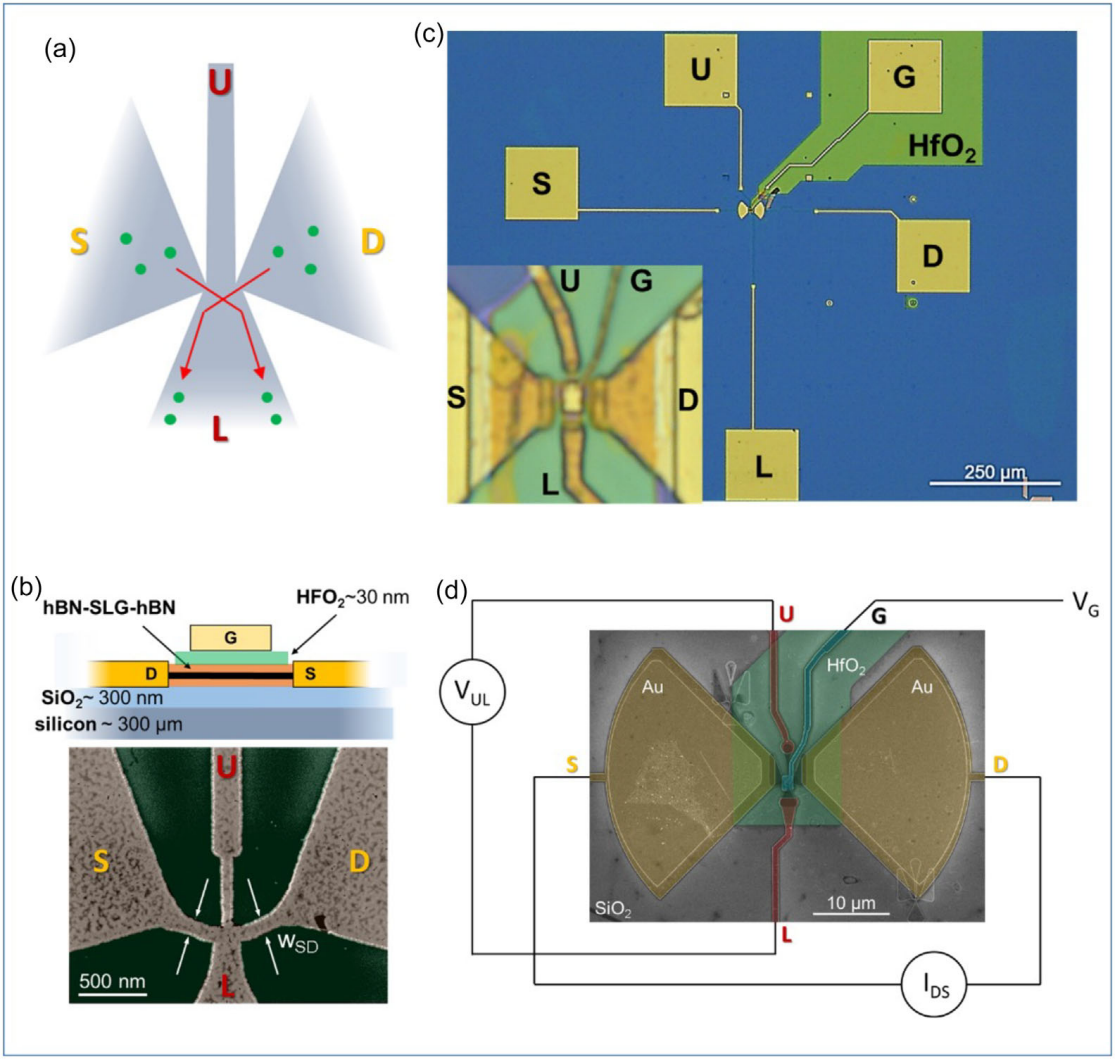
Device layout. (a) The BR geometry is characterized by an asymmetric four‐terminal junction, with electrodes labeled as source (S), drain (D), upper (U), and lower (L) contacts. Red arrows indicate the favored trajectories along which carriers propagate. (b) Schematic of device active area. SLG is encapsulated between two flakes of hBN (bottom 40 nm, top 15 nm). The LMH is capped by ∼20 nm HfO_2_ after edge‐contact fabrication. False color scanning electron microscope (SEM) image of the BR active element: a four‐terminal junction of a hBN‐SLG‐hBN heterostructure. (c) Optical images of one of the fabricated BRs. The S and D electrodes are coupled to a bow‐tie antenna resonant at 3.0 THz. (d) False color SEM image of the device and schematic diagram of a four‐probe experiment.

The conventional BR architecture consists of an asymmetric four‐terminal cross‐junction (Figure 1a). Here, rectification occurs when ACs, flowing from the source (S) to the drain (D), are transformed into DC outputs in the orthogonal direction, from the upper (U) to the lower (L) contact [2, 8, 9]. Therefore, this mechanism is primarily governed by the device's geometry, especially the two narrow, quasi‐one‐dimensional channels connecting S and D. These channels play a central role in enabling rectification, with their lateral width (*w*_SD_) being a critical factor in determining device performance. In particular, a smaller channel width leads to a stronger rectification effect [10] by confining carriers to a limited number of transverse quantum modes, denoted as *N*_SD_, within the S and D quantum point contacts (QPCs). A reduced *w*_SD_ indeed ensures that only a small number of quantum modes are occupied, which directly enhances the efficiency of the rectification process in the active region of the device [10].

Achieving high‐performance BRs requires materials with exceptionally high carrier mobility. Traditionally, semiconductor‐based heterostructures such as GaAs/AlGaAs [1] and InGaAs/InP [3] have been employed for this purpose, exhibiting carrier mobilities exceeding 100 000 cm^2^/(V×s) [3]. However, recent advancements in material synthesis and nano‐fabrication technologies, have made high‐quality single‐layer graphene (SLG) encapsulated within hexagonal boron nitride (hBN) an ideal material platform (Figure 1b), thanks to the high carrier saturation velocity (*v*_sat_ = 5.5 × 10^7^ cm s^−1^) [11], ultrafast carrier dynamics (∼ps cooling times) [12, 13], and the possibility to be synthesized or grown in the form of layered material heterostructures (LMHs), on large‐scales (∼cm^2^) [14, 15].

High‐quality LMHs composed of SLG and hBN can be fabricated using deterministic dry transfer methods [16, 17], which preserve the graphene's high carrier mobility. At room temperature, mobilities can reach up to 180 000 cm^2^ V^−1^ s^−1^, while at temperatures below 10 K, they can soar to ∼1.8 × 10^6^ cm^2^ V^−1^ s^−1^ [16]. Such exceptional mobility is critical for allowing charge carriers to move with minimal scattering over mesoscopic length scales, thereby facilitating the ballistic transport necessary for effective rectification. Furthermore, the absence of junctions eliminates the need for a threshold voltage, which is typically required to induce rectification in other devices (a noteworthy exception is represented by geometric diodes [18], which could be considered a *simplified* version of BRs). As a result, BRs can achieve zero‐threshold voltage operation, which offers several advantages. Notably, the absence of a biasing circuit simplifies the device design, reducing power consumption. Furthermore, this zero‐threshold property minimizes the flicker noise—often a significant source of noise in traditional biased detectors—thereby improving the overall detection sensitivity. Importantly, noise generated within the S–D channel does not affect the output signal, which is measured along the orthogonal U‐L direction [10]. Therefore, the output noise is primarily determined by the resistance between U and L, rather than by an intrinsic noise within the active S–D region. This enhances the signal‐to‐noise ratio (SNR) and it is beneficial to further improve the device performance in practical applications. The capability to detect low‐energy photons with high sensitivity and minimal noise is indeed a pivotal requirement for quantum sensing [19] and communication technologies [20] in the far‐infrared.

To date, BRs have shown remarkable capabilities in zero‐bias rectification, operating, efficiently, at room temperature and across a wide frequency range—from tens of gigahertz (GHz) [2, 8] to the sub‐terahertz (sub‐THz) region [9, 21]. These devices are particularly promising for applications requiring integration with planar rectennas, as their ease of integration enables seamless coupling with THz elements. Notably, recent theoretical studies [22] have demonstrated that geometrically induced ballistic electron funneling in graphene plasmonic resonators can support resonant nonlinear rectification and efficient second‐harmonic generation across the terahertz, operating without potential barriers and reinforcing the relevance of junctionless ballistic architectures for both THz detection and upconversion.

Present technologies for high‐sensitivity cryogenic detection with two‐dimensional materials include hot‐electron bolometers based on superconductor‐graphene‐superconductor (SGS) junctions that can achieve noise‐equivalent powers (NEP) as low as 30 zW×Hz^−1/2^ [23], nano‐calorimeters [24] based on magic‐angle twisted bilayer graphene (MATBG) and tunnel field‐effect transistors based on dual‐gated BLG, coupled with broadband THz antennas, showing NEPs of ∼30 fW×Hz^−1/2^ at ∼100 GHz [25] and ∼1 pW×Hz^−1/2^ at ∼3 THz [26], albeit with power dynamic ranges often limited with respect to those achievable in a BR and with difficulties in implementing scalable matrices. Recently, THz‐enhanced thermoelectricity in Moiré superlattices has been leveraged to achieve NEP < 1 pW×Hz^−1/2^ in the sub‐THz range [27], and giant THz photoresistance has been observed in MATBG [28], demonstrating that 2D Moiré systems can be engineered to explore new physical boundaries for THz receivers, offering promising opportunities for sensing applications.

Here, we conceive and engineer THz photodetectors (PDs) based on the BR mechanism, operating at high terahertz frequencies (∼3 THz), a band fully unexplored, so far, for this class of devices. The rectifying element is a high‐quality SLG channel encapsulated in hBN, integrated into planar bow‐tie THz antennas, having dimensions designed in resonance with the 3 THz field. We investigate how the geometrical width of the BR (*w*_SD_) affects the PD's performance and systematically study the performance as a function of gate voltage and impinging optical power. Remarkably, the devices show noise equivalent powers <100 pW×Hz^−1/2^ and linear operation over a power dynamic range of four‐orders of magnitude, from ∼10 nW, up to ∼1 mW (setup limited), a power range where most available technologies—for example, superconductive bolometers—suffer from responsivity saturation [29]. This performance can allow graphene‐based BRs to extend their frequency range into higher THz bands, opening up new possibilities for a wide range of applications, from high‐speed communications [20, 30], to advanced sensing technologies [31], to quantum noise applications [32, 33].

### 1.1 Device Schematics

The graphene‐based BRs have been fabricated adopting high‐quality hBN‐SLG‐hBN heterostructures. We developed reproducible reactive ion etching (RIE) recipes to establish low‐resistance (∼1 kΩ μm) electrical edge contacts on the stacks, which are instrumental for low‐temperature ballistic transport over distances longer than 15 μm [34] (further details are provided in the Methods section and in the Supporting Information ‐ S1). The device fabrication proceeded by subsequent steps of electron beam lithography (EBL) and deposition of thin layers of metals or oxide to realize a four‐terminal device, which is depicted in Figure 1a–c. In order to study how the BR geometry affects its performance, we realized two sets of devices, the first one having *w*_SD_ = 100 nm (sample A) and the second one having *w*_SD_ = 40 nm (sample B).

For electrical characterization, samples were wire‐bonded and mounted on a helium‐flow optical cryostat (Janis Technologies) with controlled heat‐sink temperature varying from 4.2 K to 300 K. The cryostat allows illuminating the sample with THz light through a high‐density polyethylene (HDPE) window (4 mm thick, transmission 58 ± 2%). The experimental setup consists of multiple *dc* source meters (SMU – Keithley2400), which allow us to perform two‐terminal and four‐terminal measurements. A schematic diagram representing the four‐terminal transport characterization is shown in Figure 1d: a current is applied between the S and D electrodes (*I*_SD_) and the voltage difference generated between U and L (*V*_UL_) is recorded as a function of the voltage applied to the top‐gate electrode (*V*_G_). We measured the resistance (*R*_DS_) as a function of *V*_G_ for one of the fabricated devices.

## 2 Results and Discussion

**FIGURE 2:**
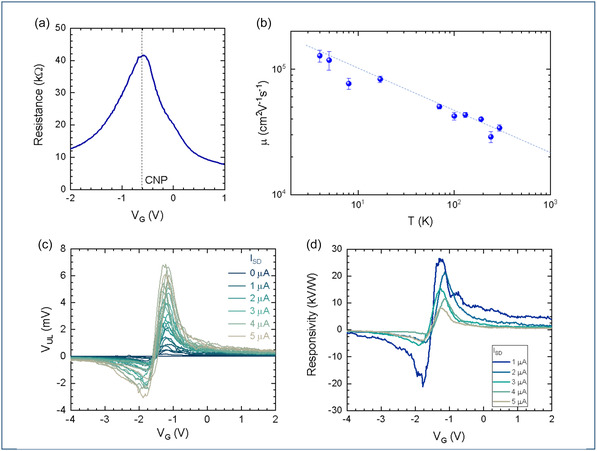
Electrical characterization of sample B. (a) Two‐terminal electrical characterization at room temperature: source‐drain resistance measured as a function of *V*_G_. (b) Two terminals (S‐D) carrier mobility measured as a function of device operating temperature, *μ* increases from 30 000 to >100 000 cm^2^/V·s when the heat‐sink temperature is reduced from 300 k to 4 K. The dashed blue line is a guide for the eye. Error bars are retrieved from the fits to the *R*_DS_ vs *V*_G_ curves. (c) Four‐terminals *V*_UL_ measured as a function of *V*_G_ for different values of *I*_SD_. Specifically, *I*_SD_ was increased stepwise from 0 to 5 μA with an increment of 0.2 μA (i.e., 0, 0.2, 0.4,…, and 5 μA). The output amplitude increases with *I*_SD_, and it is consistent with the ballistic rectification mechanism (*V*_UL_ ∝ *I*_SD_^2^). A peak conversion efficiency *η*_VI_ = 1300 V/A is obtained for *V*_G_ = −1 V. (d) Responsivity as a function of *V*_G_, calculated from the curves in (c). Measurements in a, c, d are performed at room temperature.

The gate voltage (*V*_G_) dependence on the channel resistance (*R*_DS_), measured at room temperature in sample B, is shown in Figure 2a. Similar results, for sample A, are reported in the Supporting Information—S2. From this measurement we can extract the field‐effect mobility (*μ*_FE_) for electrons and holes, the contact resistance (*R*_0_) and the residual carrier density (*n*_0_), by using the fitting function *R*_DS_ = *R*_0_ + (*L*_c_/*W*_c_) × (1/*n*_2d_ × *e *× *μ*_FE_), where *L*_c_ and *W*_c_ are the length and width of the gated area, respectively, and *e* is the elementary charge. The quantity *n*_2d_ is the gate‐dependant carrier density, given by *n*_2d_ = [*n*_0_^2^ + (*C*_g_/*e* (*V*_G_ − *V*_CNP_))^2^]^1/2^, *C*_g_ is the gate capacitance per unit area, and *V*_CNP_ is the charge neutrality point (CNP). We retrieve a *μ*_FE_ ranging from ∼20 000 cm^2^ V^−1^ s^−1^ at room‐temperature to >10^5^ cm^2^ V^−1^ s^−1^ at a heat‐sink temperature *T* < 8 K, corresponding to an electron mean‐free‐path of ∼2 μm [35]. Figure 2b shows the trend of *μ* as a function of T for sample B, with *w*_SD_= 40 nm. We note that the resistance of sample B is ∼2 times larger than the resistance of sample A (*w*_SD_ = 100 nm) near the CNP.

In order to test and assess the BR behavior, we performed four‐terminal measurements (see Figure 1d), by applying a current *I*_DS_ and measuring *V*_UL_ in dc. Figure 2c shows the results obtained, at room temperature, for sample B, whereas the results for sample A are reported in the Supporting Information ‐ S2. Near CNP the rectification mechanism is maximally efficient [9], with *V*_UL_ negative in the hole transport regime (*V*_G_ < *V*_CNP_) and positive in the electron transport regime (*V*_G_ > *V*_CNP_), in agreement with the theory [10]. *V*_UL_ grows with increasing *I*_DS_. In this regard, we can define two figures of merit: the voltage‐current conversion efficiency (*η*_VI_, units of V/A), which quantifies the amount of rectified voltage *V*_UL_ obtained per unit input current (*I*_DS_), and the voltage responsivity, *R*_V_ = *V*_UL_/(*I*_DS_^2^ *R*_DS_), which describes the *dc* rectified voltage per unit of input power (units of V/W). A plot of *R*_V_ as a function of *V*_G_ for sample B, at different excitation currents, is displayed in Figure 2d. Remarkably, *R*_V_ progressively decreases for higher input powers, suggesting the presence of a saturation mechanism in the ballistic rectification effect, that we attribute to a gradual transition to diffusive transport at high current densities [36, 37].

As expected from the theory of BRs [10, 38], according to which the rectified voltage decreases as a function of the geometrical parameter *w*_SD_ (see Supporting Information for further details), we obtain smaller *η*_VI_ and *R*_V_ for device A, which has a wider source‐drain quantum point contact: *η*_VI_(A) ∼ 70 V/A, and *η*_VI_(B) ∼ 1000 V/A, and the maximum values of *R*_V_ are 160 V/W and 20 kV/W for samples A and B, respectively.

The optical response of the detectors was subsequently characterized by using a quantum cascade laser (QCL) as THz source. The QCL is operated at a heat‐sink temperature of ∼30 K within a Stirling cryostat (Ricor, K535), which is fitted with a HDPE output window. It is driven by a pulsed waveform with a repetition frequency of 40 kHz and a 4% duty cycle, corresponding to an on‐state duration of 1 μs per pulse. This pulse waveform is further modulated by a slower envelope square wave at 1.333 kHz, which serves as a reference signal for lock‐in amplification during small signal detection. Under these conditions, the QCL average output power can be tuned from 0 to ∼1 mW. The 3 THz radiation (wavelength, *λ* = 100 μm) is focused onto the detector using two THz‐transparent TPX (poly‐4‐methylpentene‐1) lenses. The intensity distribution in the focal plane is characterized by an elliptical spot with major axis 300 μm long and minor axis 210 μm long, resulting in a beam spot area *A*_spot_ = 5 × 10^4^ μm^2^.

**FIGURE 3:**
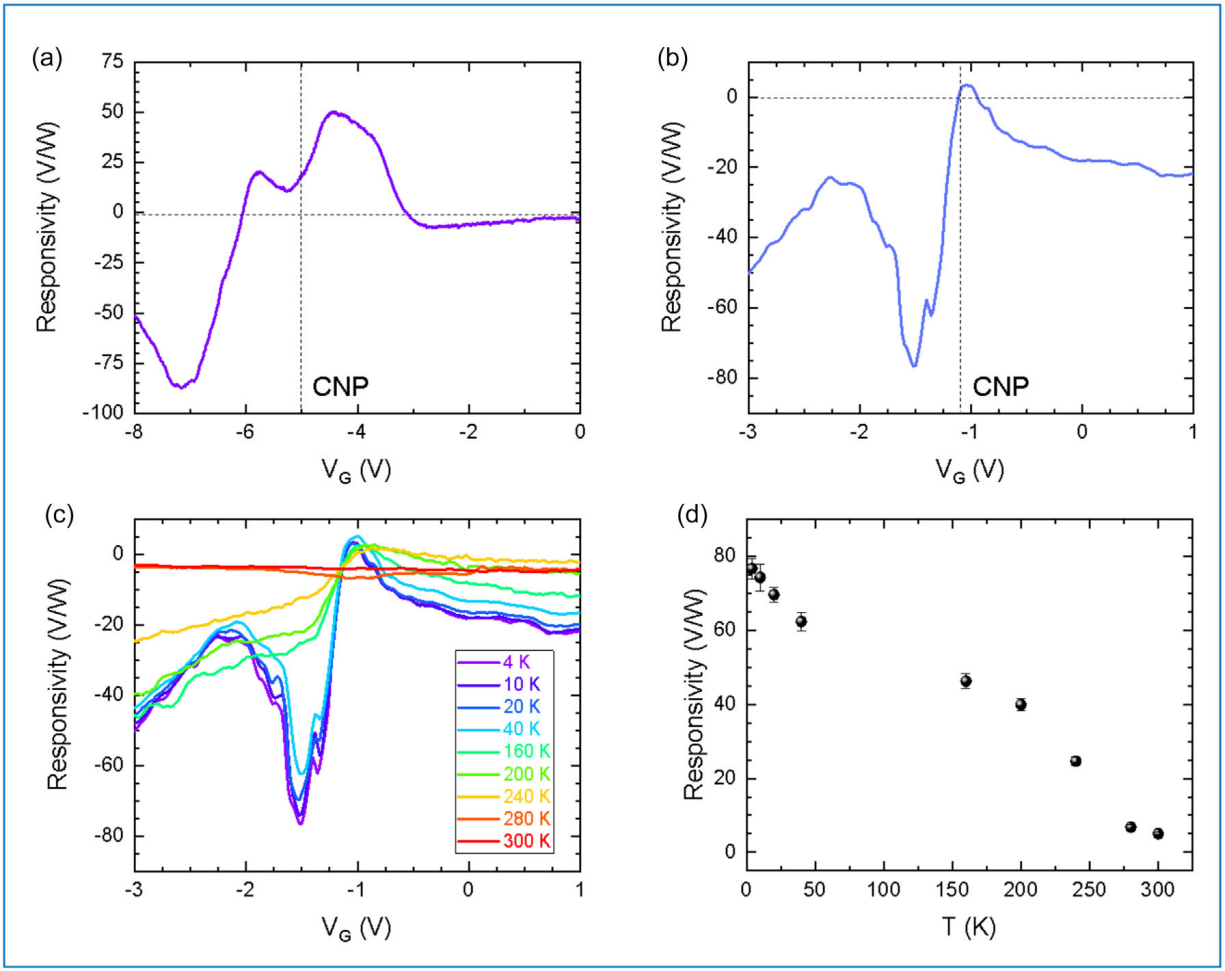
Optical characterization. (a,b) *R*_V_ plot as a function of *V*_G_, measured at 4 K, for samples A and B, respectively. (c) *R*_V_ curves as a function of *V*_G_, measured for sample B at different temperatures. (d) Maximum values of *R*_V_, from the curves in (c), plotted as a function of heat‐sink temperature. Error bars are evaluated as the standard deviation obtained on multiple *V*_G_ sweeps.

In order to isolate the THz‐induced photovoltage, Δ*u*_UL_, along the upper–lower direction (Figure 1a), we used the differential mode of a low‐noise voltage preamplifier (DLinstruments, model 1201, gain *γ* = 1000), where the U and L electrodes can be connected to two independent inputs. This configuration allows us to separate the UL reading from the electrical ground of the experiment, reducing the noise level by a factor ∼10 with respect to a standard *voltage‐to‐ground* readout (further details are reported in the Supporting Information—S4). The S and D electrodes were kept floating during the experiment and no bias was applied. The pre‐amplified signal is then measured by a lock‐in (Stanford Research 830), which is referenced to the 1333 kHz square wave modulation. Δ*u*_UL_ is estimated from the photovoltage recorded with the lock‐in, *V*_LI_, using the formula Δ*u*_UL_ = 2.2 × *V*_LI_/*γ*, where the factor 2.2 accounts for the lock‐in measuring the root mean square value of the fundamental sine wave Fourier component of the square wave envelope [39]. We evaluate the THz responsivity of the devised BRs as the ratio between the photovoltage Δ*u*_UL_ and the optical power coupled to the device. Specifically, we consider the detector effective area, *A*_eff_, to be limited by diffraction: *A*_eff_ = *λ* ^2^/4. With this assumption [26, 40], the power impinging on the detector is given by *P*_d_ = *P*_L_ × *A*_spot_/*A*_eff_, where *P*_L_ is the laser power transmitted through the cryostat HDPE input window, and *R*_V_ can be estimated as *R*_V_ = Δ*u*_UL_/*P*_d_. Figure 3a,b show the measured *R*_V_ as a function of *V*_G_ for devices A and B, respectively, at *T* = 4 K. For both devices, as expected for ballistic rectification, the response is negative for p‐type doping and positive for *n*‐type doping, with a small negative offset for both of them, potentially related to a photo‐thermoelectric contribution [41]. We note a small shift of *V*_CNP_ to more negative values, for both devices, during the optical measurements. Remarkably, despite the conversion efficiency *η*_VI_ is >10 times larger for device B, with narrower source‐drain QPCs, the THz *R*_V_ is similar for both BRs. We ascribe this result to the different high‐frequency response of the two structures: a wider *w*_DS_ results in higher transmission for THz‐induced *ac* currents between the S and D electrodes, which degrades the performance of sample B at high frequencies [42, 43]. We complement the optical characterization by analyzing the influence of the heat‐sink temperature on the optical response of sample B. Figure 3c shows *R*_V_ vs *V*_G_ recorded by varying the temperature in the 4K–300 K range. Interestingly, the sharp responsivity peak at *V*_G_ = −1.5 V becomes visible only below 40 K. We attribute this feature to an increased ballistic rectification near CNP at low temperature, as a consequence of the improved carrier mobility (Figure 2b) and reduced phonon scattering [44]. Figure 3d shows how the maximum responsivity decreases with temperature, dropping from 77 V/W at 4 K to 6 V/W at room temperature.

**FIGURE 4:**
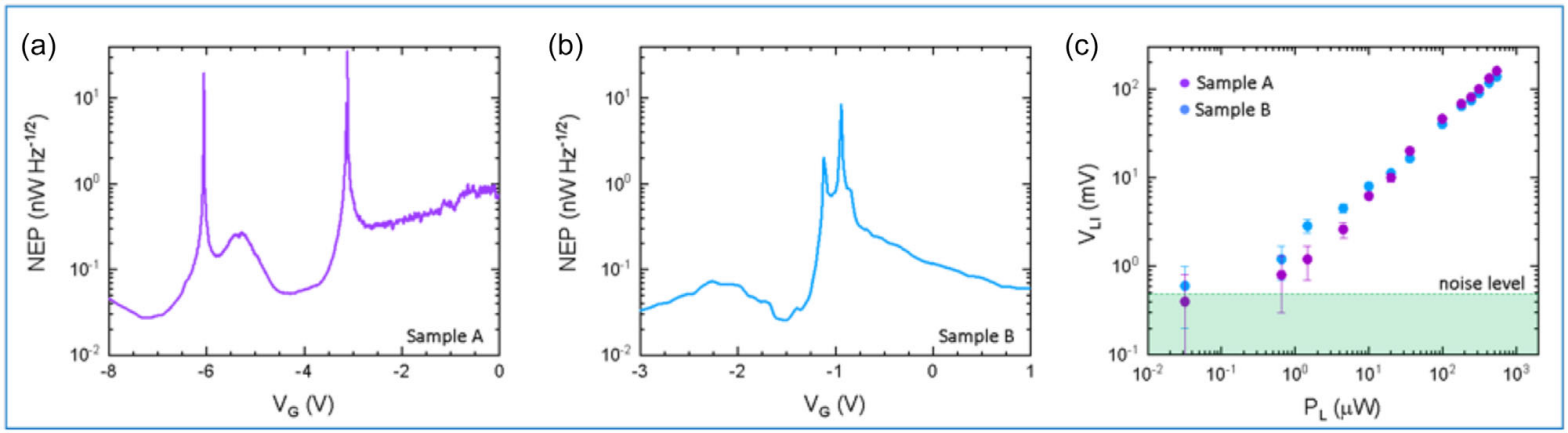
Noise equivalent power. (a,b) NEP plot as a function of *V*_G_, measured at 4 K, for samples A and B, respectively. (c) Lock‐in photovoltage plotted as a function of the laser power in log–log scale for the two investigated samples. The green shaded area represents the experimental noise floor when the lock‐in integration time is set to 100 ms. The vertical error bars are the root–mean‐square deviations of the measured *V*_LI_.

We subsequently characterize two key performance metrics of the detectors: the NEP and the minimum detectable power (MDP). These parameters provide insight into the sensitivity and detection limits of the devices under test. The NEP is defined as the amount of incident optical power required for the detector to produce a SNR of 1 in a 1 Hz output bandwidth. It quantifies the weakest optical signal that the detector can reliably distinguish from noise. NEP is evaluated as the ratio between the voltage (or current) noise spectral density (NSD) and the voltage (or current) responsivity of the detector: NEP = *R*_V_/*N*_V_, where *N*_V_ is the voltage NSD (in V/Hz^1/2^). In our analysis, we assume that the dominant source of noise is due to thermal fluctuations in the device, allowing us to estimate *N*_V_ using the Johnson‐Nyquist noise model: *N*_V_ = (4*k*_B_TR)^1/2^, where *k*_B_ is the Boltzmann constant. Figure 4a,b display the NEP as a function of *V*_G_ for samples A and B, respectively. For both of them, we obtain minimum NEP values between 20 and 30 pWHz^−1/2^. In contrast to NEP, the MDP is determined experimentally by directly measuring the dependance of the detector's output signal on varying levels of incident optical power. This is made possible by the inherent tunability of the THz QCL source, whose output power can be adjusted over several orders of magnitude by changing the applied bias voltage. This measurement allows us to infer the power dynamic range of a photodetector, with an upper limit set by the maximum average power emitted by the QCL (≈550 μV) in the Stirling cooler. Figure 4c shows the recorded lock‐in voltage (*V*_LI_) readings as a function of the power sent to the input window of the optical cryostat. The MDP is taken as the lowest power level at which a measurable signal is distinguishable from the noise floor. From this analysis, we find that sample A exhibits an MDP of ∼100 nW, while sample B achieves a significantly lower value of 30 nW, indicating improved sensitivity in the latter and a (setup limited) dynamic range larger than four orders of magnitude.

An important observation from these measurements is the distinct power‐dependent photoresponse observed in the two devices, which provides insight into how ballistic rectification behaves in the deep‐THz regime. By fitting the data in Figure 4c, with a power‐law function of the form *V*_LI_ ∼ *P*_L_^*α*^, we obtain *α*_A_ = 0.81 ± 0.02 and *α*_A_ = 0.75 ± 0.02. Both exponents indicate a sublinear photoresponse; however, the more pronounced deviation from linearity in sample B suggests that narrower QPCs are more susceptible to saturation effects under high optical power. This is consistent with the expected nonlinearity in nanoscale structures [22], which is due to carrier heating, space‐charge effects, and velocity saturation [45], which become increasingly relevant at high optical fields and high frequencies. Interestingly, despite its stronger sublinear response, sample B shows a lower MDP. This suggests that narrower QPCs may saturate more easily, consistently with enhanced current crowding and reduced phase space for ballistic transport, but simultaneously exhibit higher responsivities at low excitation powers. This observation highlights a key trade‐off, intrinsic to ballistic rectifiers operating at THz frequencies: while narrower constrictions enhance rectification efficiency and sensitivity at low incident power, they also accelerate the transition to nonlinear transport under strong excitation. These findings demonstrate that even limited variations in device geometry can significantly influence carrier dynamics and rectification efficiency at ∼3 THz, where the interplay between ballistic transport, THz‐driven carrier heating, and device impedance becomes critical. As such, the present results provide experimentally grounded design guidelines for optimizing graphene‐based BRs in the deep‐THz regime, complementing earlier lower‐frequency studies and theoretical analyses.

## 3 Conclusions

In summary, we have experimentally demonstrated graphene‐based BRs operating in a technologically relevant frequency range, in which quantum cascade lasers operate. Utilizing a 3.0 THz QCL and adopting lock‐in detection, we measure responsivities of ∼100 V/W, with NEP values of 20–30 pW/Hz^1/2^ and MDP of 30 nW.

Remarkably, the BR mechanism in graphene offers a unique combination of broadband operation, room‐temperature compatibility, and large dynamic range, which are difficult to achieve simultaneously with traditional semiconductor or bolometric THz detectors. The observed geometry‐dependent sublinear response provides direct experimental evidence that carrier dynamics and rectification efficiency in the deep‐THz regime are strongly influenced by nanoscale constrictions, revealing a trade‐off between sensitivity and linearity.

While this study does not aim to perform an exhaustive parametric exploration of device geometry, it establishes that ballistic rectification remains robust at multi‐THz frequencies and identifies key design considerations for future optimization. These results therefore complement prior low‐frequency experimental studies and recent theoretical work on nonlinear ballistic transport, by extending graphene‐based ballistic rectification into the deep‐THz domain, with competitive performance and practical relevance. Compared to the current state of the art, this technology offers a compelling route toward highly sensitive, fast, and integrable detectors suitable for emerging applications in spectroscopy, imaging, and wireless communications within the THz frequency domain.

## 4 Methods

### 4.1 Device Fabrication

To fabricate the hBN/SLG/hBN heterostructure, we employed a dry‐transfer technique. Initially, hexagonal boron nitride (hBN) flakes were mechanically exfoliated onto polydimethylsiloxane (PDMS) substrates and identified under an optical microscope. A square poly(propylene) carbonate (PPC)/PDMS/glass stamp was then used to pick up a suitable top hBN flake. The SLG, previously exfoliated onto a SiO_2_/Si substrate, was aligned beneath the hBN flake using a precision transfer system and laminated at 100°C to form the top hBN/SLG stack. The assembled stack was subsequently cleaned with acetone and isopropyl alcohol (IPA), followed by vacuum annealing at 300°C for 90 min. To prepare the bottom hBN layer, a separate SiO_2_/Si substrate was cleaned using the same procedure as for graphene. hBN flakes were exfoliated onto Nitto tape, thinned by repeated peeling, and transferred onto the cleaned substrate. After resting for 2 min to promote adhesion, the blue tape was slowly peeled off over ∼30 s. Clean, thick hBN flakes were identified under an optical microscope. The previously annealed hBN/SLG stack was then picked up using a dome‐shaped PPC/PDMS/glass stamp, aligned over the bottom hBN, and released to complete the full hBN/SLG/hBN heterostructure. A final cleaning step using acetone and IPA was performed, followed by a second vacuum anneal at 300°C for 90 min. All flake pickup procedures were performed using either square or dome‐shaped PPC/PDMS/glass stamps, with transfer and release temperatures set at 40°C and 100°C, respectively.

**FIGURE 5:**
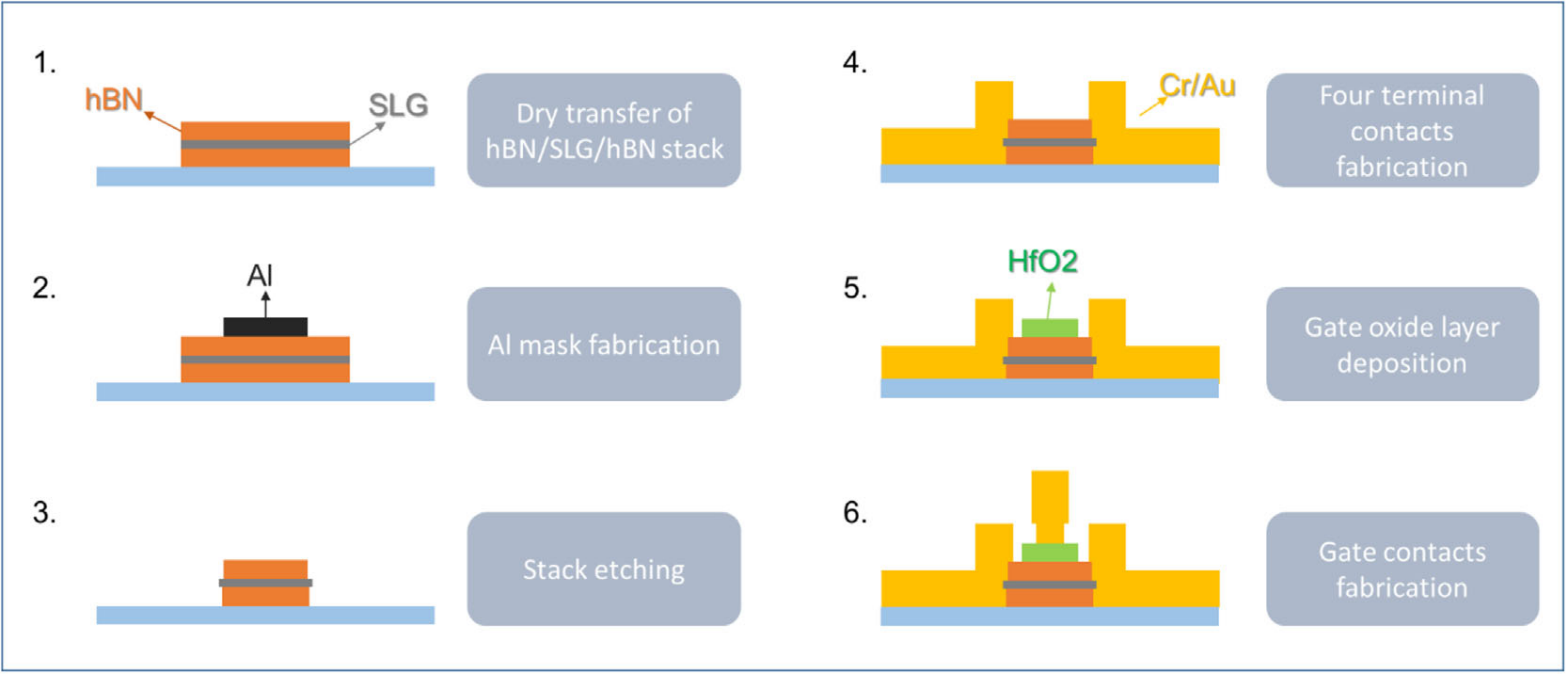
Schematic representation of the different fabrication steps.

BR devices were then fabricated from the assembled heterostructures, as illustrated in Figure 5. Alignment marks were patterned using either electron‐beam lithography (EBL) or laser direct writing, followed by thermal evaporation of Cr/Au (5/50 nm) and standard lift‐off. To shape the core rectifier channel, a 20 nm‐thick Al layer was used as a hard mask. This was defined using EBL, evaporation, and lift‐off. After mask preparation, unprotected regions of the hBN/SLG/hBN stack were etched using RIE, with a gas mixture of CF_4_ (20 sccm) and O_2_ (4 sccm) under 12 W RF power (bias: −118 V). After etching, the Al mask was removed using MF319 developer, followed by a deionized water rinse and N_2_ blow‐drying. Four Cr/Au electrodes (5/150 nm), along with integrated antenna structures, were patterned using EBL, metal evaporation, and lift‐off. A 20 nm HfO_2_ gate dielectric was deposited by atomic layer deposition (ALD), with its lateral geometry defined via EBL and lift‐off. Finally, Cr/Au top gates (5/100 nm) were fabricated using the same process sequence.

## Supporting Information

Additional supporting information can be found online in the Supporting Information section. **Supporting Fig. S1**: Mechanical exfoliation of single‐layer‐graphene. (a) Stacked Raman spectra of the as‐exfoliated SLG layer on Si/SiO_2_ substrate, and of the hBN‐SLG‐hBN heterostructure. (b‐e) Optical images of the mechanically exfoliated SLG. **Supporting Fig. S2**: Electrical characterization of Sample A. (a) Channel resistance as a function of V_G_ at two different temperatures. (b) Four‐terminal measurement of V_UL_ *vs* V_G_, obtained with I_DS_ = 300 nA and T = 120 K. (c) Responsivity calculated from the curve in (b). **Supporting Fig. S3**: Dependance of the rectification voltage, V_UL_, as a function of the source and drain QPC width, *w*_SD_. The inset shows a schematic of the center of the BR channel, with the indication of the angle *θ*_0_. **Supporting Fig. S4**: Characterization of additional sample, C, *w*_SD_ = 100 nm. (a) Dependnce of the voltage, V_UL_, as a function of source‐drain current and gate voltage, measured at T = 4 K. The range of V_G_ where the device operates as a ballistic rectifier is near V_G_ = −1.0 V, which corresponds to the CNP value. (b) V_UL_, as a function of source‐drain current, for V_G_ = −1.0 V and V_G_ = −1.2 V. (c‐d) Low temperature responsivity and signal‐to‐noise ratio (for 100 μW input optical power) measured in different device configurations (e). The largest responsivity is observed in configuration C#3, the typical one for BRs. Configuration C#4 also shows a large SNR. All configurations display a responsivity peak near the CNP.

## Author Contributions

**Lili Shi**: data curation (equal), investigation (lead). **Leonardo Viti**: conceptualization (equal), data curation (equal), investigation (supporting), writing – original draft (equal). **Kenji Watanabe**: methodology (supporting). **Takashi Taniguchi**: investigation (supporting). **Miriam Serena Vitiello**: data curation (supporting), funding acquisition (lead), methodology (equal), project administration (lead), resources (lead), writing – original draft (equal), writing – review & editing (lead).

## Funding

This work was supported by the ERC POC Terascan (101157731), Italian Ministry of University and Research (PNRR PE0000023‐NQSTI).

## Conflicts of Interest

The authors declare no conflicts of interest.

## Code Availability

The codes and simulation files that support the plots and data analysis within this paper are available from the corresponding author upon reasonable request.

## Supporting information

Supplementary Material

## Data Availability

The data that support the plots within this paper and other finding of this study are available from the corresponding authors upon reasonable request.
