# Supplementary material for: Nanoscale Graphene‐Based Ballistic Rectifiers for Detection of High Terahertz Frequency Optical Signals
Source: Small Sci. 2026 Mar 29;6(4):e202500654. doi: 10.1002/smsc.202500654 (PMC13124028; doi:10.1002/smsc.202500654)
Supplement: Supplementary file 1 — Supplementary Material [file SMSC-6-e202500654-s001.pdf]

Supporting Information

Nanoscale graphene-based ballistic rectifiers for detection of high terahertz frequency optical signals

Lili Shi[1], Leonardo Viti[1], Kenji Watanabe[2], Takashi Taniguchi[2], Miriam S. Vitiello[1]
[1]*NEST, CNR-NANO and Scuola Normale Superiore, 56127, Pisa, Italy*
[2]*National Institute for Materials Science, 1-1 Namiki, Tsukuba, 305-0044, Japan*

S1 - Mechanical exfoliation of SLG

A 2-inch high-resistivity silicon wafers (resistivity > 20 kΩ·cm) coated with a 300 nm thermally grown SiO_2 layer was initially adopted for transfer. A layer of AR-P 679.04 photoresist was spin-coated onto the wafers, which were then diced into smaller chips for processing. The chips were cleaned sequentially in acetone and isopropanol (IPA) to remove organic contaminants, followed by nitrogen blow-drying. To activate the substrate surface, the chips were loaded into a reactive ion etching (RIE) system and exposed to an oxygen plasma (100 sccm O_2 flow, 20 W RF power, bias voltage: –120 V) for 9 minutes. During this step, graphene flakes were prepared via mechanical exfoliation. A bulk graphite crystal was pressed onto Nitto blue tape and repeatedly peeled to thin the material. Through sequential folding and peeling, the graphite layer thickness was reduced while achieving a broader spatial distribution of flakes.

Once the substrates were prepared, suitable single-layer graphene (SLG) regions were identified by their characteristic light-grey appearance on the blue tape. The selected SLG region and the target SiO_2/Si chip were mounted onto a glass slide. Immediately after RIE treatment, the flake-bearing portion of the tape was laminated onto the cleaned substrate and gently pressed into contact. The sample was then baked on a hotplate at 100 °C for 2 minutes to improve adhesion. After cooling for 2 minutes under a directed nitrogen gas flow (applied to both sides of the chip), the tape was carefully and rapidly peeled off, leaving SLG flakes adhered to the substrate. This transfer process routinely yields high-quality SLG flakes with lateral dimensions exceeding 2500 μm^2, suitable for device fabrication.

Figure S1(a) compares the Raman spectra of the same single-layer graphene (SLG) before and after hBN encapsulation. For the exfoliated SLG, the characteristic G peak at ~1580 cm^{-1} and the symmetric 2D peak at ~2700 cm^{-1} with higher intensity than the G peak confirm its monolayer nature. After encapsulation with hBN, the graphene-related G and 2D peaks are well preserved, indicating that the encapsulation process does not introduce noticeable structural degradation. In addition, an $E_{2}g$ phonon mode of hBN emerges at ~1366 cm^{-1}, confirming the successful formation of the hBN–SLG–hBN heterostructure. The absence of an observable D peak in both spectra

suggests a low defect density in the same SLG flake, demonstrating that the hBN encapsulation effectively maintains the high crystalline quality of graphene.

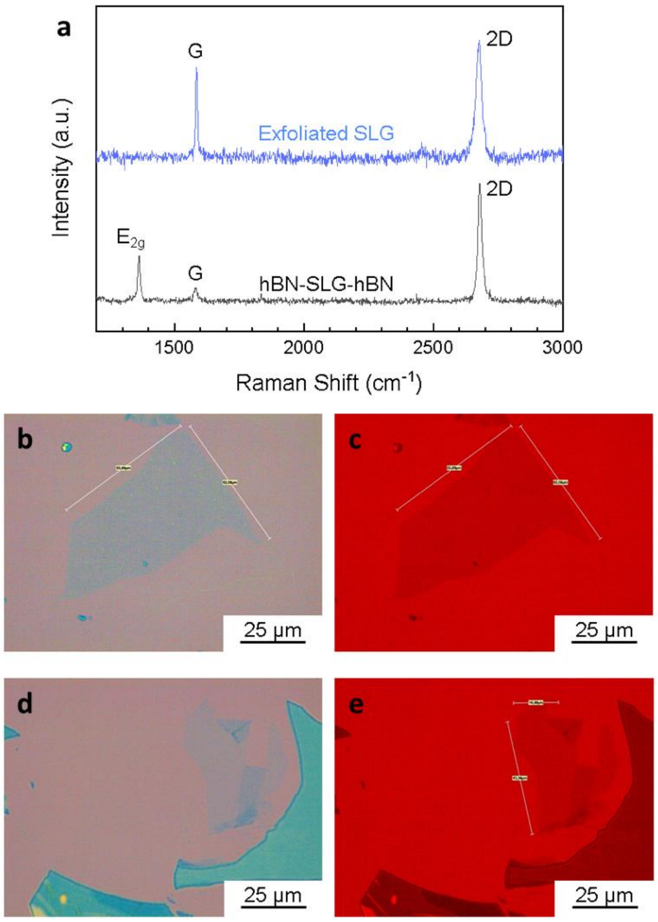

Figure S1. Mechanical exfoliation of single-layer-graphene. (a) Stacked Raman spectra of the as-exfoliated SLG layer on Si/SiO_2 substrate, and of the hBN-SLG-hBN heterostructure. (b-e) Optical images of the mechanically exfoliated SLG.

S2 - Electrical characterization of Sample A

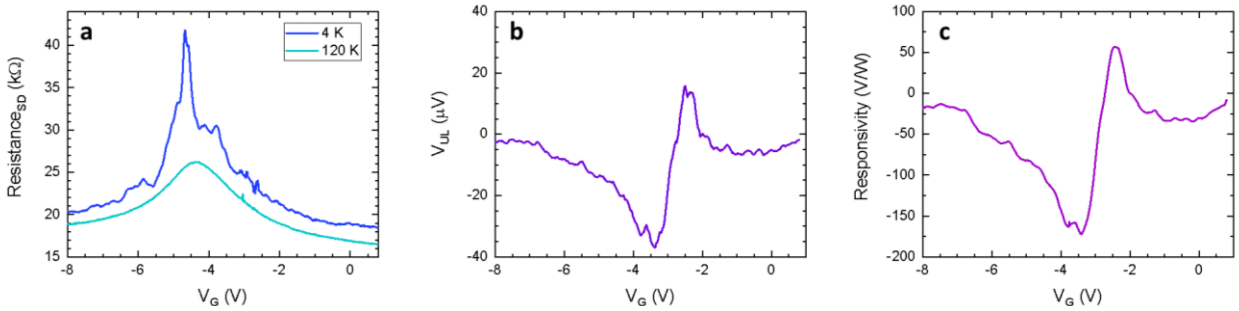

Figure S2. Electrical characterization of Sample A. (a) Channel resistance as a function of V_G at two different temperatures. (b) Four-terminal measurement of V_{UL} *vs* V_G, obtained with I_{DS} = 300 nA and T = 120 K. (c) Responsivity calculated from the curve in (b).

S3 –Dependence on nano-channel geometry

The dependence of the rectification signal (V_{UL}) generated by a ballistic rectifier on its geometric parameters has been extensively investigated in a recent paper by J. Brownless and co-workers (ref. [10]). The theory predicts that the rectification signal decreases when the widthw_{SD} of the source and drain quantum point contacts (QPC) is reduced. The rectification mechanism is the governed by the expression:

$$V_{UL} = \frac{h\sqrt{\pi}}{4e^3 v_f w_{SD}} \frac{\sin(2\theta_0)}{2w_U - w_{SD}(1-\sin(\theta_0))^2} \frac{I_{SD}^2}{n^{3/2}}$$

Where h is the Planck's constant, e is the elementary charge, v_f is the Fermi velocity in graphene, θ_0 is the angle between the QPC normal and the scatterer edge (see Figure S3), w_U is the width of the *upper* contact, I_{SD} is the source-drain current and n is the carrier density. By using this expression, we reproduce the trend displayed in Figure S3, where Sample A, with w_{SD} = 100, and Sample B, withw_{SD} = 40 nm, are marked by a red and a yellow star, respectively.

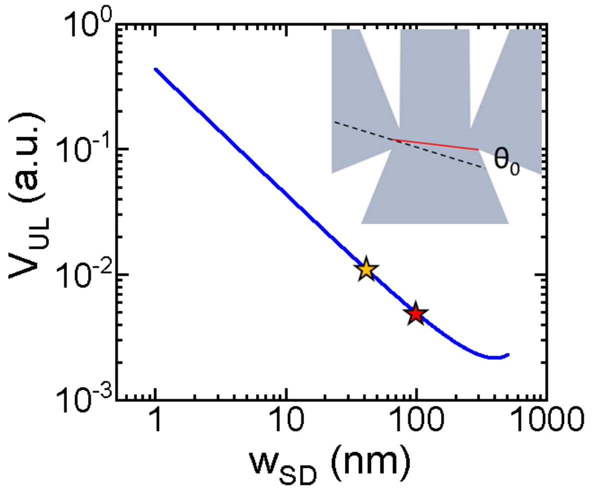

Figure S3. Dependence of the rectification voltage, V_{UL}, as a function of the source and drain QPC width, w_{SD}. The inset shows a schematic of the center of the BR channel, with the indication of the angle θ_0.

S4 – Additional devices and measurement configurations

To further assess device-to-device reproducibility and optimize the readout scheme, we fabricated and characterized an additional graphene-based ballistic rectifier (Sample C) with source–drain constriction width w_{SD}= 100 nm, identical to Sample A discussed in the main text.

The electrical response of Sample C is shown in Figure S4. The device exhibits clear ballistic rectification within a finite gate voltage window, −1.6 V <V_G< −0.6 V (Figure S4a,b). This interval is centred around the charge neutrality point, V_{CNP}=−1.0 V, where the carrier density is minimized and the ballistic contribution to transport becomes dominant. Within this gate voltage range, the responsivity displays a pronounced peak (Figure S4c), consistent with the onset of ballistic rectification. The coincidence between the rectification regime and the responsivity maximum

confirms that the detection mechanism is governed by geometry-induced ballistic carrier trajectories, as discussed in the main text. To evaluate the influence of the readout scheme, we investigated four distinct measurement configurations, schematically illustrated in Figure S4e. In all configurations, the responsivity maximum occurs for $V_G \approx V_{CNP}$, demonstrating that the rectification mechanism is intrinsic to the device architecture rather than measurement-specific.Among the tested configurations, configuration C#3 — where the photovoltage is measured between the upper (U) and lower (L) electrodes, corresponding to the standard readout geometry for ballistic rectifiers — exhibits the highest responsivity and the best signal-to-noise ratio (SNR > 100 for an incident optical power of 100 μW). Configuration C#4 also yields a comparatively large SNR (Figure S4d). The consistency of the gate-dependent peak position across all configurations further supports the robustness of the ballistic rectification mechanism in independently fabricated devices.

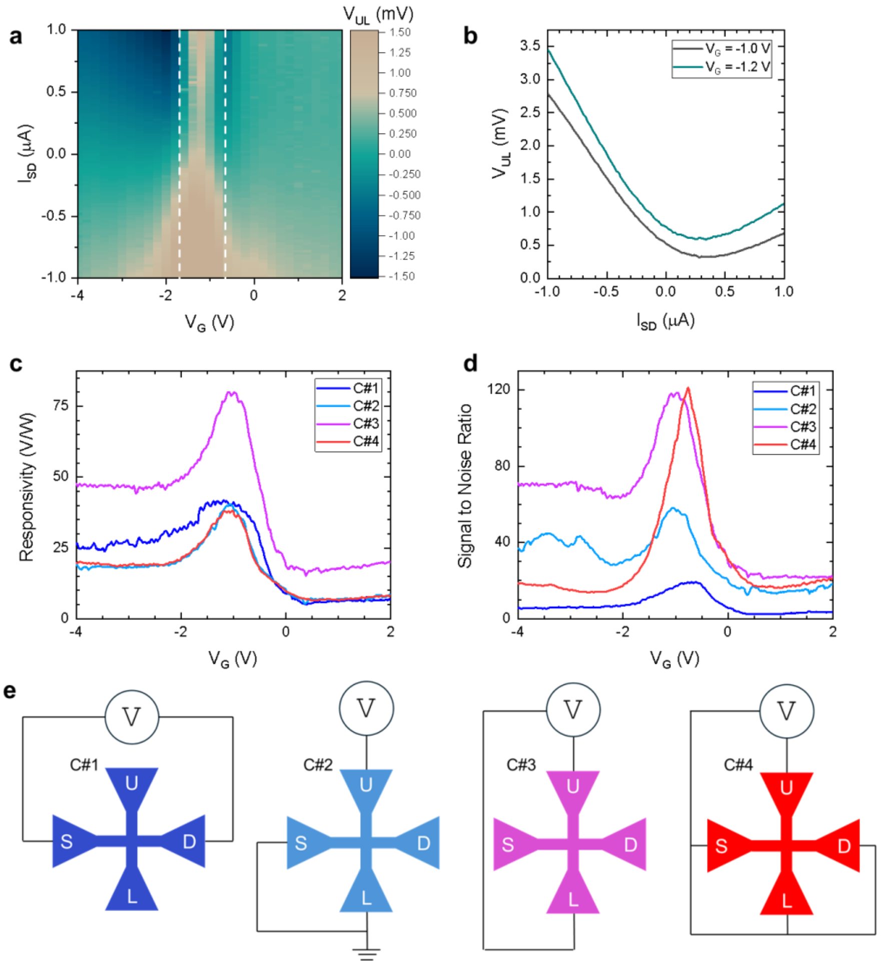

Figure S4. Characterization of additional sample, C, w_{SD} = 100 nm. (a) Dependence of the voltage, V_{UL}, as a function of source-drain current and gate voltage, measured at T = 4 K. The range of V_G where the device operates as a ballistic rectifier is near $V_G = -1.0$ V, which corresponds to the CNP value. (b) V_{UL}, as a function of source-drain current, for $V_G = -1.0$ V and $V_G = -1.2$ V. (c-d) Low temperature responsivity and signal-to-noise ratio (for 100 μW input optical power) measured in different device configurations (e). The largest responsivity is observed in configuration C#3, the typical one for BRs. Configuration C#4 also shows a large SNR. All configurations display a responsivity peak near the charge neutrality point.
